# Knowledge of type 2 diabetic patients about their condition in Kimpese Hospital diabetic clinic, Democratic Republic of the Congo

**DOI:** 10.4102/phcfm.v9i1.1385

**Published:** 2017-09-20

**Authors:** Patrick N. Ntontolo, Philippe N. Lukanu, Gboyega A. Ogunbanjo, Jean-Pierre L. Fina, Léon N.M. Kintaudi

**Affiliations:** 1 Department of Family Medicine and Primary Health care, Protestant University of Congo, Democratic Republic of the Congo; 2 Institut Médical Evangélique (IME), Democratic Republic of the Congo; 3 Department of Family Medicine and Primary Health Care, Sefako Makgatho Health Sciences University (SMU), South Africa

## Abstract

**Background:**

Diabetes mellitus is a worldwide increasing health problem of which type 2 diabetes is the most prevalent. Previously considered as a problem of industrialised countries, diabetes is currently a huge concern in developing countries and the Democratic Republic of the Congo (DRC) is one of the sub-Saharan countries with a high prevalence rate of diabetes. Deficit of knowledge has already been shown to be one of the barriers preventing diabetic patients from controlling their disease.

**Objectives:**

This study aimed to assess the knowledge of type 2 diabetic patients seen at the Institut Médical Evangélique (IME) Kimpese Hospital diabetic clinic, DRC, and the factors associated with their knowledge.

**Methods:**

A cross-sectional study involving 184 respondents was conducted at the diabetic clinic of the IME Kimpese Hospital, DRC. We administered a pre-tested questionnaire. Out of a total of 10, scores of < 5, 5 to < 7, and ≥ 7 were classified as ‘poor knowledge’, ‘moderate knowledge’ and ‘good knowledge’, respectively, according to expert consensus. All statistical tests were performed using *p* < 0.05 as the level of statistical significance.

**Results:**

The mean age of respondents was 57.5 years (s.d. ± 1.4, ranging from 40 to 83 years), with 56% being male. The mean diabetes knowledge score was poor: 3.2 out of a total of 10 (s.d. ± 1.7), with the range between 0.2 and 7.7. The majority of respondents (72.3%) had poor general knowledge about diabetes mellitus. Respondents also scored poorly in areas of the causes (35.6%), risk factors (39.3%), clinical features (34.9%), complications (20.5%) and management (42.4%) of diabetes mellitus. Using the student *t*-test analysis, it was found that age (*p* = 0.001), gender (*p* = 0.002), educational level (*p* = 0.007) and duration of disease (*p* = 0.032) were significantly associated with poor knowledge of diabetes mellitus.

**Conclusions:**

Knowledge of diabetes mellitus among type 2 diabetic patients seen at our setting was poor. Areas of deficiency and factors associated with knowledge of diabetes were identified. Our findings suggest the need for a health education intervention programme for our diabetic patients.

## Introduction

Diabetes mellitus (DM) is an increasing health problem globally. The estimated number of 415 million people living with the disease is predicted to rise to 642 million by 2040, while the related mortality is estimated to result in one death every 6 s.^1^ Type 2 diabetes mellitus (T2DM) is known to be the most prevalent type of this condition.^1,2^ Diabetes was previously considered as a problem of industrialised countries, but is currently a huge concern in developing countries, especially in sub-Saharan Africa.^3^ With a prevalence of 6.1%, the Democratic Republic of the Congo (DRC) is estimated to be the fifth sub-Saharan country in terms of people living with DM after Nigeria, South Africa, Ethiopia and United Republic of Tanzania.^1^ A study at the Institut Médical Evangélique (IME) Kimpese Hospital, DRC, reported a prevalence of 5.5%, and 12% of these diabetic patients had died by the time of follow-up six years later.^4^ Therefore, decreasing the burden of this chronic disease and delaying its complications through glycaemic control should be a high priority.^5^ Poor and inadequate glycaemic control among patients with T2DM is acknowledged to be a substantial health problem and a major risk factor for the development of diabetes complications. This requires much effort by health care professionals, the patient and family as well. Although the benefits of strict glycaemic control are evident, especially in reducing microvascular and macrovascular complications,^6^ it has been reported that about 60% of diabetic patients do not achieve the recommended glycaemic control target.^7,8,9,10^ Several studies have explored the factors that may be associated with poor glycaemic control. The outcome was that various factors were associated with poor glycaemic control, namely inadequate medication, deteriorating DM owing to the duration of the disease and poor adherence enhanced by lack of or poor patients’ knowledge about their condition.^11,12^ Given that literature about the knowledge of patients with T2DM in the Kongo-Central Province and the rural DRC was scarce, a need to evaluate their knowledge of DM was considered appropriate. This study aimed to assess the knowledge of type 2 diabetic patients seen at IME Kimpese diabetic clinic, DRC, and the factors associated with their knowledge.

## Methods

### Design

This was a cross-sectional study involving 184 respondents.

### Setting

The study was conducted among previously diagnosed patients with T2DM attending the Outpatient Diabetic Clinic of IME Kimpese Hospital, DRC. Kimpese is a city located 220 km west of Kinshasa, the capital city of the DRC. This hospital is one of the biggest hospitals in the Kongo-Central Province of the DRC with a capacity of 400 beds. Diabetes clinical services are provided on Tuesdays to approximately 25 patients who are given appointments every month, except those necessitating closer follow-up or three-monthly visits. Patients attending this hospital reside in various places and some are from neighbouring countries.

### Study population and sample

A total of 255 DM patients attended the diabetic clinic from January 2013 to July 2013. Eligibility criteria for the study were as follows: all type 2 diabetic patients registered for a follow-up at the diabetic clinic at IME Kimpese Hospital, DRC, should be at least 40 years old, diagnosed with T2DM for at least three months prior to the study and must provide consent to participate in the study. Those who did not meet one or more of the above criteria (e.g. type 1 diabetic patients and less than 40 years of age) and especially those who did not consent to participate were not included in the study. A convenience sample of 184 patients with T2DM met the inclusion criteria and consented to participate in the study.

### Data collection

The 21-items questionnaire used in this study was inspired from a modified version of the Michigan Diabetes Research and Training Centre’s Brief Diabetes Knowledge Test.^13^ This was combined with the 24-item Diabetes Knowledge Questionnaire that was shown to have a good internal consistency and construct validity in the Starr County Diabetes Education Study.^14^ A pilot study was carried out with 10 patients who met the inclusion criteria for a final adjustment of the questionnaire. The principal researcher and one trained nurse collected data from the type 2 diabetic patients who consented to the study on each diabetic clinic day. All recruited type 2 diabetic patients completed the questionnaires in a private consulting room in the clinic. It was a self-administered questionnaire for those who were literate and researcher-administered for those who were illiterate. The first part of the questionnaire consisted of nine items related to age, gender, tribe, ethnic group, residence, educational level, marital status, occupation and anthropometric data (i.e. weight and height). The second part also consisted of nine questions and was related to the diagnosis, causes, complications and management of diabetes. The last three questions tried to find out the sources of previous education of the respondent on his or her condition. Once the study questionnaire was completed, the researcher checked for any missing data and the correctness of data before the subjects left the clinic.

### Data analysis

After the interview, a scoring system was developed for each component. Each correct answer was given a score of 1 and each wrong or ‘I do not know’ answer was given a zero. An approximate or unsure reported year was given a half score (0.5). For multiple answers, a score of 1 was divided by the total number of correct answers. The maximum score of nine (considered as raw score) was obtained by summing the number of correct responses. Thus, the final scores were converted to out of 10 from raw scores by using the following formula: (raw score × 10)/9. Regarding diabetes knowledge, three categories were defined on the basis of the score obtained by each participant: poor knowledge for less than 5 out of 10, moderate knowledge for 5–6.9, and good knowledge for 7–10. The body mass index (BMI) of each participant was calculated by using the following formula: weight (kg)/height(m)^2^, and participants were classified as underweight, normal weight, overweight and obese according to the World Health Organization international classification of adult BMI. Statistical analysis was performed using the Epi-Info^™^ version 3.5.1. Univariate analyses were carried out on demographic and other variables to obtain standard descriptive statistics (e.g. mean, standard deviation, ratio, frequency and percentage) according to the objectives of the study. The mean age in years, male to female ratio, the general diabetes knowledge score and percentage of the correct answers in specific areas (e.g. cause, symptoms, risk factors, management and complications) were also calculated. The occupation and the social position reported by the respondents (i.e. employer, self-employed or employee) helped to categorise them. Then, three socioeconomic classes were considered: upper class, middle class and lower class. The student *t*-test analysis was used to assess the influence of different variables on the scores obtained. This included the influence of sociodemographic and health characteristics of diabetic patients to their general and specific knowledge. All significance tests were two-tailed and all variables with a *p*-value of < 0.05 were considered statistically significant with a 95% of confidence interval (95% CI).

### Ethical considerations

The Medunsa Research and Ethics Committee South Africa approved the study (MREC/M/19/2011: PG), followed by permission from the hospital head of Institut Médical Evangélique Kimpese Hospital, DRC. All participants received information about the aim and objectives of the study, and signed the consent form prior to completing the questionnaire. Confidentiality and anonymity of the respondents were maintained by group data analysis and no personal identifiers were used.

## Results

Among the 184 participants, 103 (56%; CI: 48.49–63.27) were male, with the female to male sex ratio of 1:1.27. The mean age was 57.5 ± 1.4 years, ranging from 40 to 83 years. According to the occupation, the majority of participants (84.2%) were in the lower class, 7.6% were from the middle class and 8.2% were from the upper class. In terms of the highest level of education attained, 6.5% were illiterate, 24.5% reported to have completed only primary school, 58.7% had a secondary school education and 10.3% achieved university education (high school education included). The majority of the participants (73.9%) were married, 17.9% widowed, 3.9% divorced, 1.6% separated and 2.7% were single (Table 1).

**TABLE 1:** Associations between sociodemographic variables and diabetes knowledge; distribution of body mass index among participants; association of body mass index with gender.

Variables	*n*	%	Mean score	95%CI	*t*	*p*
Gender						
Male	103	56	3.5	3.2–3.9	3.12	0.002
Female	81	44	2.8	2.4–3.1	-	-
Age						
40–44	18	9.8	3.4	2.6–4.3	1.39	0.165
45–49	17	9.2	3.3	2.3–4.2	1.74	0.084
50–54	32	17.4	2.7	2.1–3.6	3.28	0.001
55–59	33	17.9	4.2	3.6–4.9	-	-
60–64	47	25.5	3.1	2.6–3.6	2.75	0.007
65–69	17	9.2	2.6	1.6–3.2	2.89	0.004
≥ 70	20	11	2.4	1.6–3.2	3.53	0.001
Socioeconomic class						
Upper class	15	8.2	3.5	2.3–4.6	-	-
Intermediate class	14	7.6	3.6	2.6–4.5	1.41	0.162
Lower class	155	84.2	3.1	2.8–3.4	1.94	0.054
Education						
Illiterate	12	6.5	1.9	0.9–2.9	2.72	0.007
Primary school	45	24.5	2.9	2.4–3.5	1.45	0.149
Secondary school	108	58.7	3.2	2.9–6.2	0.99	0.324
University	19	10.3	3.8	2.8–4.6	-	-
Marital status						
Single	5	2.7	3.6	1.9–5.2	0.90	0.370
Married	136	73.9	3.2	2.8–3.5	1.67	0.097
Divorced	7	3.9	4.7	2.9–6.3	-	-
Separated	3	1.6	3.4	1.4–5.4	0.94	0.350
Widowed	33	17.9	2.7	2.1–3.3	2.18	0.031
BMI						
Underweight^a^	7	3.8	2.7	1.5–4.0	0.89	0.375
Normal weight^b^	95	51.6	3.1	2.7–3.5	0.81	0.419
Overweight^c^	60	32.6	3.4	2.9–3.8	-	-
Obese^d^	22	12	3.1	2.4–3.9	0.43	0.671

BMI, body mass index; CI, confidence interval.

a Underweight (BMI < 18.5 kg/m^2^).

b Normal weight (BMI 18.5–24.9 kg/m^2^).

c Overweight (BMI 25–29.9 kg/m^2^).

d Obese (BMI ≥ 30 kg/m^2^).

The duration of the disease varied among respondents from 11 months to 38 years. Patients who had the condition less than 10 years represented the majority of participants (77%) (Table 2).

**TABLE 2:** Diabetes knowledge related to duration of the disease.

Duration (year)	*n*	%	Mean score	95%CI	*t*	*p*
< 10	142	77	3.0	2.67–3.28	2.17	0.032
10–19	33	18	3.8	3.11–4.42		
≥ 20	9	5	4.0	2.78–5.12	0.27	0.788

CI, confidence interval.

Seven respondents had a BMI less than 18.5 kg/m^2^; 95 respondents (51.6%) were classified with a normal BMI of 18.5–24.9 kg/m^2^; 60 respondents (32.6%) were classified as overweight with a BMI of 25.0–29.9 kg/m^2^; and 22 respondents (12.0%) were classified as obese with a BMI of 30 kg/m^2^ or more (Table 1). The mean BMI was 24.90 ± 0.62 kg/m^2^ (CI: 24.30–25.52). Female respondents had higher mean BMI than male respondents (26.0 ±1.0 kg/m^2^ vs 23.9 ± 0.7 kg/m^2^, respectively) and the difference was statistically significant (*p* = 0.0011).

### Knowledge of diabetes mellitus

The average knowledge score of the diabetic patients was 3.2/10 (s.d. = 1.7) and ranged from 0.2 to 7.7. Close to three-quarters of respondents (72.3%) had poor general knowledge about the disease, whereas 38 (20.7%) had moderate knowledge and 13 (7.0%) had good knowledge about diabetes, respectively. Respondents scored poorer on specific knowledge questions. The mean percentage score was 34.9% for clinical manifestations, 35.6% for causes of DM, 20.5% for complications, 42.4% for the management of the condition, and 39.3% for risk and aggravating factors (Table 4).

**TABLE 3:** Distribution of general knowledge of participants.

General knowledge group	*n* = 184	%
Poor knowledge	133	72.3
Moderate knowledge	38	20.7
Good knowledge	13	7.0

Using the student *t*-test, the analysis of knowledge (mean score) correlated to gender has shown that men were more informed about diabetes than women (*p* = 0.002). Data related to age demonstrated that respondents in the age group of 55–59 years scored better than others did. When compared to the youngest group of 40–44 years, the difference was not statistically significant (*p* = 0.165). However, when compared to older groups, there was a significant statistical difference and the age group of 70 years and above scored the worst (*p* = 0.001). The result showed no statistical difference between the upper and the lower socioeconomic class (*p* = 0.054). Four educational levels were compared, illiteracy (no education), primary school, secondary school and university level, and the results showed that the knowledge scores increased with increasing educational level. Compared to university group, the illiterate group was associated with poor knowledge (*p* = 0.007), but there was no significant statistical difference with the primary and the secondary levels. The marital status comprised five categories: single participants, married, divorced, separated and widowed. All five categories had poor knowledge as displayed in Table 1. Although the divorced respondents seemed to be more knowledgeable, there was no significant statistical difference when compared to other groups except the widowed category (*p* = 0.031).

The duration of the disease was distributed in three groups. The largest proportion of the patients (77%) reported to have been diagnosed less than 10 years ago. The first group (less than 10 years) scored poorer than the two other patient groups in which the disease has been diagnosed more than 10 years earlier. Indeed, there was a progressive increase in the performance of diabetic patients correlated to the increase in the number of years post-diagnosis. The mean score of patients who were diagnosed less than 10 years earlier was 3.0, the mean score of those diagnosed 10–19 years previously was 3.8 and the mean score of those diagnosed 20 years ago or earlier was 4.0.

## Discussion

Age has been demonstrated as a risk factor for DM^15^ and most people affected by T2DM in developed countries are over 60 years old.^16^ In contrast, the majority of individuals with DM in Africa are less than 60 years of age with the highest proportion of DM (43.2%) in those aged 40–59 years.^17^ The findings of our study are similar to the above data, as the majority of participants (147/184; 79.8%) were less than 65 years of age. When considering the threshold of 60 years, the proportion of participants aged less than 60 years remains higher than those 60 years and above (54.3% vs 45.7%). The predicted increase was observed when comparing the proportion of 45.7% in our study to 18.8% estimated in the global estimates of diabetes prevalence study by Guariguata et al. for people over 60 years old.^17^

Although this study was not designed to establish the significance of sex differences among diabetic patients, there were more men than women. This trend is consistent with the prevalence of DM reported by Muyer and colleagues in their study, where men were slightly more than women with 5.5% (95% CI: 3.9%–7.6%) and 4.4% (95% CI: 3.3%–5.8%) respectively.^18^ Also, Longo-Mbenza et al. reported the same observation, but the difference between men (53 or 12.2%) and women (40 or 10.7%) was not statistically significant.^19^

Regarding BMI, obesity has been shown to be one of the major risk factors in the development of T2DM.^20^ Respondents presenting overweight and obesity jointly constitute a non-negligible proportion (44.6%) in this study. A similar worrying proportion has already been documented in one study from South Africa, where the prevalence of overweight and obesity was the highest (39.6% and 37.2%, respectively) in respondents in the age group 51–60 years.^21^

This study has shown that general knowledge about DM was extremely limited among the participants as the majority (72.3%) of them presented poor knowledge as they scored less than 5 out of 10. This status is consistent with many other previous studies.^22,23,24^ In contrast, Fezeu et al.^25^ found in their study conducted in Cameroon that 80% of subjects scored better than the total mean score. This might be because the study was conducted in an area where health promotion and health education on diabetes have been intensively delivered for the past four years. A case-control study carried out in eight hospitals of Kinshasa reported poor knowledge in 66.2% of diabetic patients.^26^ This proportion is less than the one found in our study which took place in a semirural milieu. Deepa et al.^27^ reported similar observations in their study conducted in four regions of India and where, overall, urban residents had higher awareness rates (58.4%) compared to rural residents (36.8%).

Respondents scored poorer in specific areas of diabetes as reported in Table 4. Regarding clinical manifestations, we noted that respondents had limited knowledge and misinterpretations of the presenting signs and symptoms of diabetes. Indeed, the mean score percentage for this area was 34.9%. This tends to influence the nature of care sought and the timing of care seeking. Similar observations were made by Al Shafaee et al., among Omani population where inadequate public awareness and knowledge about diabetes symptoms might explain the lack of early care seeking and diagnosis for diabetes.^28^ On the other hand, when the signs and symptoms do not fit into the existing traditional health beliefs, the disease is easily associated with supernatural powers owing to the lack of an adequate socially acceptable explanation. Concerning the cause of DM, the mean score percentage was unsatisfactory (35.6%). This gap might presuppose that diabetic patients have particular beliefs and lay perceptions of DM from which emerge ideas of witchcraft. This may partially explain the reason why some diabetics seek traditional healers’ services rather than orthodox medical services. Although this study did not focus on the attitude and behaviour of participants, this phenomenon may represent the iceberg of misconception that affects glycaemic control in many African communities, as reported in two studies conducted in Cameroon.^29,30^ In one of the studies, slightly over half of the respondents (53%) associated the causes of type 2 diabetes with excessive sugar intake.^29^Another finding in which the respondents failed was on risk factors, and the mean score percentage was 39.3%. Large disparities were observed in their responses. The main risk factors reported by respondents were weight gain (18.5%), excessive sugar intake (52%) and fatty foods (35%). Only 1.6% reported the ‘lack of exercise’ as a risk factor for DM. Regarding complications, the mean score percentage was very low (20.5%). This score resulted from the fact that few respondents reported blindness (35.3%), cardiac complications (20%), kidney problems (19%), stroke (21.7%), leg ulcers (25.5%) and amputation (21.7%) as complications related to diabetes. O’Sullivan et al. reported similar results, although the awareness of cardiovascular complications was higher (53.5%) in their study.^31^ Respondents scored better in the management of DM (42.4%), and a possible explanation for this relative performance is that this topic is the one diabetic patients usually focus on during consultation with the health care provider.

**TABLE 4:** Distribution of mean percentage score of specific knowledge from all participants.

Specific knowledge area	Mean percentage score		Total
	Correct responses	Wrong responses	
Clinical manifestation	34.9	65.1	100
Cause of diabetes	35.6	64.4	100
Complications	20.5	79.5	100
Management	42.4	57.6	100
Risk factors	39.3	60.7	100

Association of knowledge with gender in our study showed that men were more knowledgeable about diabetes than women (*p* = 0.002). This finding is consistent with the results from a study conducted in the general population of Cameroon.^25^ Another recent study from Bangladesh reported similar findings.^32^ This may be explained by the fact that literacy levels in women are generally lower than those of their male counterparts in developing countries. Families tended to educate male children at the expense of female children. However, Lemes Dos Santos et al. found that Brazilian women tended to achieve better knowledge scores about diabetes than men in their study population.^33^

Correlation of knowledge with age showed that the age group 55–59 years scored better than others, but the significant statistical difference was observed only in older groups. The age group of 70 years and more scored worse, and when compared to the high-performance group, the difference was statistically significant (*p* = 0.001).This finding is corroborated by the study conducted in Bangladesh.^31^ The authors found that people in older age groups showed significantly poorer knowledge about diabetes in six of the items, compared to the people in younger age groups. The significantly higher level of knowledge in younger age groups was primarily explained by their higher levels of education. The trend from the current study seems to be consistent with the above consideration when we consider the declining trend from the mean score of 3.4 to 2.4 for the first age group and the seventh group, respectively (Table 1). While a number of studies revealed no association between age and knowledge of diabetes,^34,35^ a study from Switzerland^36^ reported an opposite tendency as it found that increasing age was positively associated with awareness of diabetes among participants presenting with T2DM. The high level of literacy in most developed countries might be the reason for this controversy.

It is known that there are strong correlations between occupation and income. Several studies have established the negative association of low income with knowledge of DM. Al-Adsani et al. reported that participants with limited family income had significantly lower knowledge scores among Kuwati adults with T2DM.^37^ In our study, the lower class tended to score worse than the upper and middle classes, respectively. However, there was no significant difference among them as far as diabetes knowledge was concerned.

We observed from our results that there was a definite association between educational level and diabetes knowledge. When compared to those who achieved university education, the illiterate group demonstrated poor knowledge about diabetes (*p* = 0.007). The more educated the respondents, the better their knowledge scores about diabetes (Table 1). The Al-Adsani et al. study also found similar associations between limited health literacy and poorer disease knowledge.^37^ Although limited health literacy appeared to exert its influence through diabetes knowledge, Bains and Egede^38^ found that there was not any influence on diabetes self-care or medication adherence.

Regarding marital status, we found that divorced patients scored better than other groups, specifically when compared to widowed participants (*p* = 0.031). The incidence risk for this marital status as reported by Cornelis et al.^39^ could not explain the performance observed in our study. Therefore, more investigation is required to explain this observation.

The majority of respondents (77%) were diagnosed less than 10 years ago with diabetes. This group presented the poorest knowledge score compared with those whose disease was diagnosed more than 10 years ago. When comparing the mean score of the group diagnosed 10–19 years ago with the first group of less than 10 years ago, difference was found statistically significant (*p* = 0.032) (Table 2). Similar findings have been reported by Murata et al.^40^ who stated that a longer duration of the disease was associated with better knowledge.^40^ These findings are not consistent with those of West and Goldberg,^41^ who found no significant increase in knowledge scores with the number of years post-diagnosis in the Veterans’ Clinic in the USA. This contrast might be explained by the lack of established education programmes on diabetes in our context, which would have empowered the patient’s knowledge about diabetes before its development.

## Strengths and limitations

To our knowledge, this is the first study to be conducted in our setting in the DRC among diabetic patients. Although the study population interviewed was not representative of the general population, most of the sociodemographic characteristics were consistent with the literature cited.

We acknowledge the following study limitations:

- The cross-sectional nature of this study only allowed us to obtain a snap shot opportunity of information about the knowledge among type 2 diabetic patients attending IME Kimpese diabetic clinic, as we could not establish the causality of the research findings. Therefore, caution should be made in generalising our findings as patients were recruited in a hospital setting.
- Some of the data were self-reported, such as date (year) of diagnosis, which may have resulted in recall bias.
- Participants for this study were obtained by a ‘convenience’ sampling frame, potentially introducing selection bias, as patients who attended to the clinic were those who usually cared about their health.
- In the context of low-resource hospitals in rural Africa, we did not measure glycated haemoglobin (HbA1c) so that we could check association of diabetes knowledge with glycaemic control.

## Conclusion

The findings of the present study identified profound knowledge deficiencies among type 2 diabetic patients. Knowledge of DM among type 2 diabetic patients seen at our setting was poor. Areas of deficiency and factors associated with knowledge of diabetes were identified. Our findings suggest the need for an intervention health education programme for our diabetic patients.

Age and gender were shown to be associated with knowledge. Years since diagnosis of diabetes and formal education were also positively associated with diabetes knowledge. Longer duration of diabetes irrespective of educational status was associated with a higher knowledge score. In light of these findings, action must be taken now in order to increase communities’ knowledge of diabetes, especially that of patients attending IME Kimpese Hospital diabetic clinic. If no action is taken, the prevalence of diabetes will continue to increase and will ultimately become an unbearable burden for the community and country.
